# Recurrent gastrointestinal bleeding after bowel perforation surgery in immunocompetent patients: invasive gastrointestinal mucormycosis: a case report

**DOI:** 10.1186/s12879-025-11783-9

**Published:** 2025-12-09

**Authors:** Le Yang, Sheng-long Chen

**Affiliations:** Department of Critical Care Medicine, Guangdong Provincial People’s Hospital, Guangdong Academy of Medical Sciences, Southern Medical University, Guangzhou, China

**Keywords:** Gastrointestinal mucormycosis, Gastrointestinal bleeding, Invasive fungal infection, Immunocompetent patient, Endoscopic biopsy

## Abstract

A 73-year-old man was hospitalized for intestinal perforation and underwent surgery. Subsequently, he experienced recurrent gastrointestinal bleeding. A second operation revealed invasive mucormycosis—a rare but severe fungal infection of the digestive tract. Typically treated with amphotericin B, mucormycosis often occurs in immunocompromised patients; however, this patient had no history of immunosuppressive therapy or known risk factors. In cases of gastrointestinal bleeding, performing biopsies and cultures during endoscopic examinations when ulcers are detected is recommended to ensure accurate diagnosis.

**Supplementary Information:**

The online version contains supplementary material available at 10.1186/s12879-025-11783-9.

## Introduction

Gastrointestinal mucormycosis is a rare but highly lethal form of mucormycosis, accounting for approximately 7% of all mucormycosis cases [1]. Despite its rarity, it is associated with extremely high mortality rates ranging from 85% to 92% in adults. Mucormycosis itself is a life-threatening fungal infection characterized by vascular invasion and is responsible for approximately 10% of all systemic fungal infections [2]. It can invade blood vessels and the gastrointestinal wall [3], leading to severe complications such as sepsis, peritonitis, massive gastrointestinal bleeding, and intestinal perforation—common causes of death. The intestines are the most frequently affected site (71.6%), followed by the stomach (33.0%) [4]. However, only 25% of gastrointestinal mucormycosis cases are diagnosed before death [5]. 

## Case study

A 73-year-old male presented with a one-day history of acute, diffuse abdominal pain without clear onset and persistent without relief. He exhibited no fever or chills. A CT scan at a local emergency department suggested an abdominal aortic aneurysm, leading to symptomatic treatment, including pain management and blood pressure control, before transferring to our facility.

### Examination and investigations

**Fig. 1:**
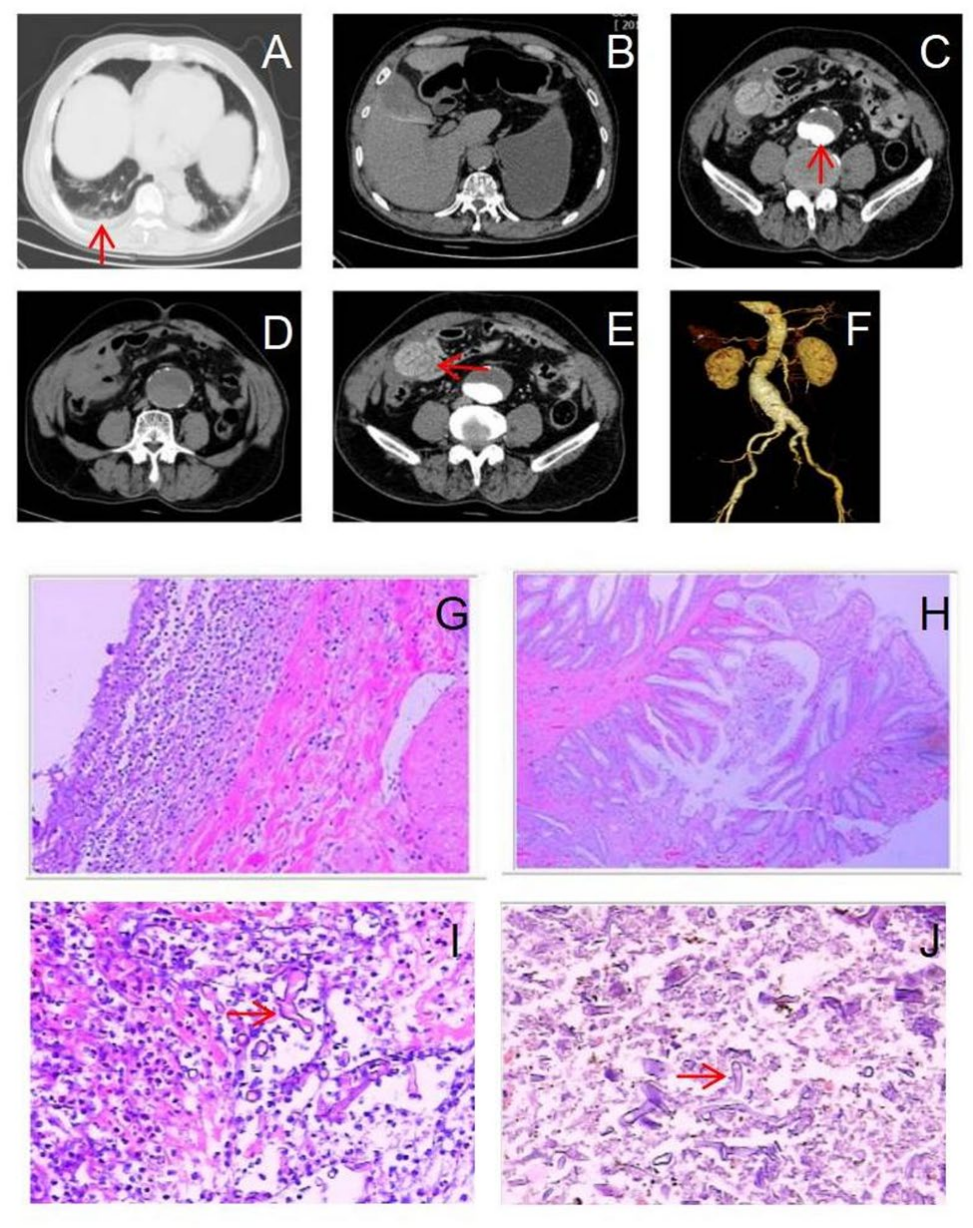
**A**-**F**: Contrast-enhanced CT at admission revealed aortic sclerosis and an infrarenal abdominal aortic aneurysm with adjacent thrombus (red arrow, Panel **C**). Additional findings included coronary artery sclerosis and a myocardial bridge in the mid-segment of the left anterior descending artery. A mild right-sided pleural effusion was also observed (red arrow, Panel **A**). Perihepatic fluid, mild pneumoperitoneum suggestive of gastrointestinal perforation, and inflammatory changes with fluid in the right lower abdomen were present, indicating possible peritonitis (red arrow, Panel **E**). Cholecystitis was also suspected. **G**-**H**: Histopathology from the first surgery showed features consistent with intestinal perforation, polypoid projections, and hyperplastic polyps. **I**-**J**: Histopathological sections from Day 32 revealed extensive ulceration involving the stomach, small intestine, colon, and spleen. Broad, ribbon-like fungal hyphae morphologically consistent with mucormycosis were observed. Special stains included Periodic Acid–Schiff (PAS), Gomori Methenamine Silver (GMS), and acid-fast staining. Fungal elements invading tissue are indicated by red arrows

Upon admission, the patient was afebrile (37 °C) with stable vital signs: heart rate of 103 bpm, respiratory rate of 21 bpm, and blood pressure of 90/51 mmHg, indicative of low perfusion state, evidenced by weak peripheral pulses. Physical examination revealed diminished breath sounds with rales in the right lung and abdominal distension with mild tenderness and a palpable pulsatile mass in the lower abdomen. Initial laboratory findings included: white blood cell count 4.9 × 10⁹/L, hemoglobin 165 g/L, glucose 9.07 mmol/L, blood urea nitrogen 9.30 mmol/L, creatinine 166 µmol/L, alanine aminotransferase 17 U/L, and total bilirubin 23.1 µmol/L. An enhanced abdominal CT scan was performed (Fig. 1, Panels A–F).

Later that day, emergency exploratory laparotomy revealed a large amount of purulent fluid in the abdominal cavity. A 1 × 1.5 cm perforation was identified at the rectosigmoid junction. A partial sigmoidectomy with temporary colostomy was performed, and the patient was transferred to the intensive care unit (ICU). Histopathological findings from the surgical specimen are shown in Fig. 1, Panels G and H. Upon ICU admission, under general anesthesia, the patient had weak peripheral pulses and signs of poor peripheral perfusion. Vital signs were stable: temperature 37 °C, pulse 103 bpm, respiratory rate 25 bpm, and blood pressure 90/60 mmHg. Lung auscultation revealed coarse breath sounds with rales, and abdominal distension was noted, accompanied by a functional drainage tube in the right lower abdominal cavity near the intestinal anastomosis site, draining dark red blood. Mild edema was present in both lower extremities.

## Day 14

The patient developed abdominal pain and passed approximately 800 mL of tarry stool through the stoma. Vital signs included a heart rate of 100 bpm and blood pressure of 96/59 mmHg. Laboratory evaluation revealed a hemoglobin level of 51 g/L, indicating gastrointestinal bleeding. Management included transfusions of red blood cells, plasma, and cryoprecipitate, along with hemostatic therapy, gastric protection using omeprazole, and fluid resuscitation. Gastrointestinal endoscopy revealed multiple ulcers in the esophagus, stomach, and colon. Ulcer biopsies were not performed due to the patient’s hemodynamic instability and coagulopathy related to active gastrointestinal bleeding.

## Day 26

Following the removal of skin staples, the patient experienced wound dehiscence with bowel exposure, necessitating emergency surgical intervention. Intraoperative findings included erosion and necrosis of the stomach and spleen, multiple perforations in both the small and large intestines, and necrosis surrounding the descending colostomy site. Surgical procedures included total gastrectomy with Roux-en-Y esophagojejunostomy, splenectomy, temporary transverse colostomy, ileal resection, ileotransverse anastomosis, and resection of the descending colon. Pathological examination revealed ulceration and fungal infection involving the stomach, small intestine, colon, and spleen, consistent with mucormycosis (Fig. 1, Panels I–J).

## Treatment and outcome

Following the diagnosis of gastrointestinal mucormycosis, intravenous amphotericin B (5 mg/day) was initiated with gradual dose escalation. However, on day 38, the patient’s family chose to discontinue antifungal therapy, given the patient’s advanced age, prolonged hospitalization, progressive physical decline, poor prognosis, and financial constraints.

## Discussion

Gastrointestinal mucormycosis (GIM) is a rare but life-threatening fungal infection characterized by extensive angioinvasion, resulting in thrombosis, tissue necrosis, gastrointestinal bleeding, and intestinal perforation. Despite its severity, GIM is frequently diagnosed late due to its nonspecific clinical manifestations and the limited diagnostic yield of early tests.

In the present case, routine hematoxylin and eosin (H&E) staining of the initial surgical specimen following sigmoid perforation did not reveal fungal elements. Consequently, special fungal stains—such as periodic acid–Schiff (PAS) and Gomori methenamine silver (GMS)—were not applied at the time, potentially contributing to the delayed diagnosis. This underscores the importance of considering early implementation of special staining techniques in patients with unexplained intestinal perforation or ulcerative lesions.

Mucormycosis typically occurs in immunocompromised individuals, including those with hematologic malignancies, solid organ transplants, or uncontrolled diabetes [5, 6]. However, recent literature has reported an increasing number of gastrointestinal mucormycosis (GIM) cases in immunocompetent patients, particularly those who are critically ill due to trauma, sepsis, or organ failure, and subsequently develop massive gastrointestinal bleeding or perforation. In our case, the patient had no history of immunosuppressive therapy, malignancy, neutropenia, or diabetes. Nonetheless, several clinical features—such as colonic perforation with diffuse peritonitis, a low white blood cell count (nadir 2.88 × 10⁹/L), and a subdued inflammatory response—may reflect a state of acquired or functional immunosuppression.

In addition, the combination of localized bowel ischemia, prolonged hospitalization, broad-spectrum antibiotic exposure, and poor nutritional status likely contributed to disruption of the intestinal mucosal barrier and impairment of host defenses, thereby facilitating fungal invasion. These factors may have acted synergistically to promote the development of invasive gastrointestinal mucormycosis in this patient.

The gold standard for diagnosing mucormycosis is histopathological confirmation, as fungal cultures often yield negative results. In many cases, definitive diagnosis relies solely on biopsy or smear analysis. Therefore, in patients with gastrointestinal bleeding or unexplained ulcerative lesions, early endoscopic biopsies and fungal staining—such as PAS or GMS—should be considered, even in the absence of overt suspicion for fungal infection.

Gastrointestinal bleeding may serve as an early yet underrecognized clinical indicator in the differential diagnosis of GIM. Invasive mucormycosis of the GI tract often presents with vague, nonspecific symptoms, and serious complications such as perforation or hemorrhage can lead to rapid deterioration, septic shock, and multi-organ failure. Early diagnosis is challenging due to subtle clinical signs and the limited sensitivity of routine tests. Thus, in patients with gastrointestinal bleeding and multiple ulcerative lesions without a clear etiology, tissue biopsy combined with fungal staining and microbial cultures is strongly recommended. These diagnostic measures are essential for prompt identification and initiation of antifungal therapy [7].

The pathophysiology of mucormycosis is defined by its aggressive angioinvasive nature, leading to tissue infarction, necrosis, and rapid progression of clinical symptoms [8]. Vascular thrombosis in infected tissues impairs antifungal drug delivery, reducing the effectiveness of pharmacological treatment alone. Therefore, successful management often requires a combination of early surgical debridement and systemic antifungal therapy.

Liposomal amphotericin B remains the first-line treatment for mucormycosis, administered at a recommended dose of 5 mg/kg/day. Compared to amphotericin B deoxycholate, the liposomal formulation offers superior safety—particularly with regard to nephrotoxicity—and enhanced tissue penetration [9]. Effective treatment strategies also involve correcting underlying risk factors and managing any contributing immunosuppressive states.

## Supplementary Information

Supplementary Material 1.

## Data Availability

The data used during the current case are available from the corresponding author on reasonable request. To protect patient confidentiality, certain identifying details have been omitted or anonymized.

## Abbreviations

CT: Computed Tomography
WBC: White Blood Cell Count
HGB: Hemoglobin
GLU: Glucose
BUN: Blood Urea Nitrogen
CREA: Creatinine
ALT: Alanine Aminotransferase
TBIL: Total Bilirubin
GIM: Gastrointestinal mucormycosis
