# Supplementary figures and images for: Recurrent gastrointestinal bleeding after bowel perforation surgery in immunocompetent patients: invasive gastrointestinal mucormycosis: a case report
Source: BMC Infect Dis. 2025 Dec 9;25:1703. doi: 10.1186/s12879-025-11783-9 (PMC12690881; doi:10.1186/s12879-025-11783-9)

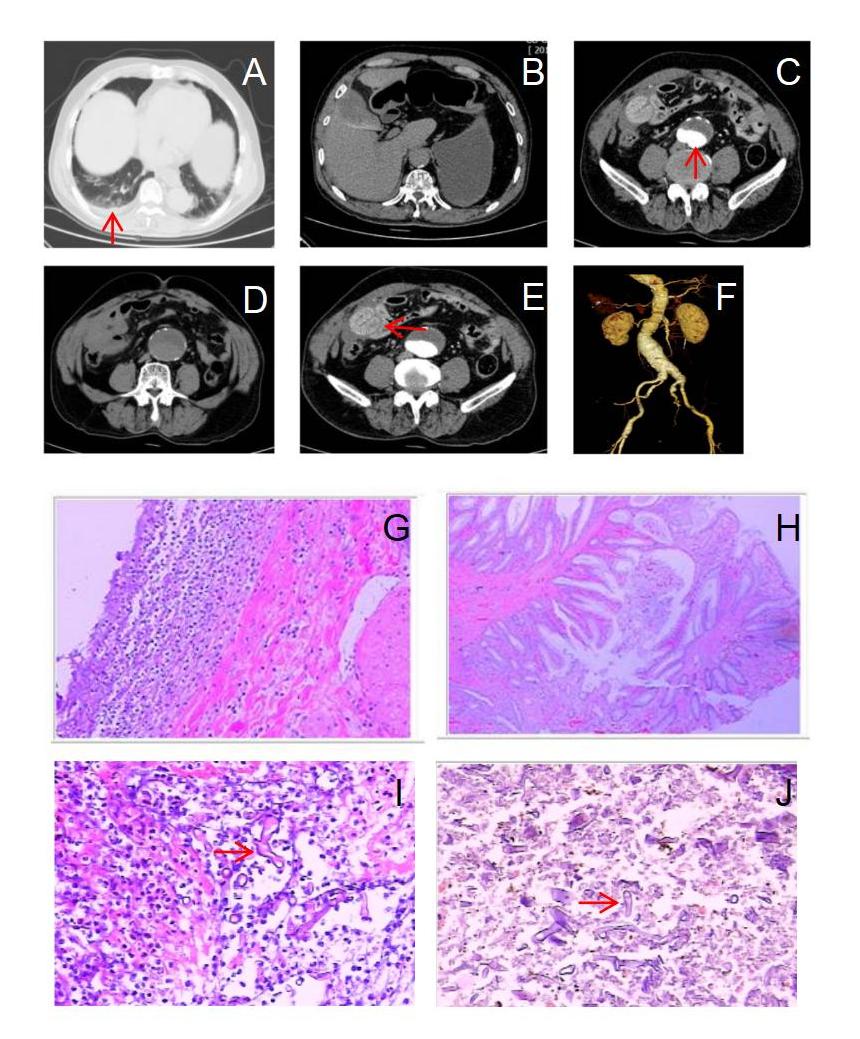

Supplement: Supplementary file 1 — Supplementary Material 1. [file 12879_2025_11783_MOESM1_ESM.jpg]
